# Process for developing rehabilitation practice recommendations for individuals with traumatic brain injury

**DOI:** 10.1186/s12883-017-0828-z

**Published:** 2017-03-20

**Authors:** Librada Callender, Rachel Brown, Simon Driver, Marie Dahdah, Ashley Collinsworth, Shahid Shafi

**Affiliations:** 1 Clinical Research Coordinator, Baylor Institute for Rehabilitation, 909 N. Washington Ave, Dallas, TX 75246 USA; 2 Clinical Research Analyst, Office of the Chief Quality Officer, Baylor Scott & White Health, Dallas, TX USA; 3 Baylor Institute for Rehabilitation, Dallas, TX USA; 4 Center for Medical Psychology, Baylor Regional Medical Center of Plano, Plano, TX USA; 5 Office of the Chief Quality Officer, Baylor Scott & White Health, Dallas, TX USA; 6 Director of Rehabilitation Research, Baylor Institute for Rehabilitation, Dallas, TX USA

**Keywords:** Traumatic brain injury, Brain injury, Rehabilitation, Delphi, Clinical practice guideline, Evidence based medicine

## Abstract

**Background:**

Attempts at measuring quality of rehabilitation care are hampered by a gap in knowledge translation of evidence-based approaches and lack of consensus on best practices. However, adoption of evidence-based best practices is needed to minimize variations and improve quality of care. Therefore, the objective of this project was to describe a process for assessing the quality of evidence of clinical practices in traumatic brain injury (TBI) rehabilitative care.

**Methods:**

A multidisciplinary team of clinicians developed discipline-specific clinical questions using the Population, Intervention, Control, Outcome process. A systematic review of the literature was conducted for each question using Pubmed, CINAHL, PsychInfo, and Allied Health Evidence databases. Team members assessed the quality, level, and applicability of evidence utilizing a modified Oxford scale, the Agency for Healthcare Research and Quality Methods Guide, and a modified version of the Grading of Recommendations, Assessment, Development, and Evaluation scale.

**Results:**

Draft recommendations for best-practice were formulated and shared with a Delphi panel of clinical representatives and stakeholders to obtain consensus.

**Conclusion:**

Evidence-based practice guidelines are essential to improve the quality of TBI rehabilitation care. By using a modified quality of evidence assessment tool, we established a process to gain consensus on practice recommendations for individuals with TBI undergoing rehabilitation.

## Background

For patients who suffer significant neurologic and functional deficits due to traumatic brain injury (TBI), inpatient rehabilitation centers provide comprehensive post-injury care that has been shown to improve functional outcomes and successful reintegration into the community [1]. The structures and processes of care during inpatient rehabilitation can highly influence patient outcomes [2, 3]. However, there is a growing recognition that there is variability in application of evidence-based guidelines, demonstrating a need for a standard treatment approach [4]. For example, amongst TBI Model System (TBIMS) rehabilitation centers, which provide a state-of-the-art multidisciplinary system of rehabilitation care [5], significant differences in risk-adjusted functional outcomes of TBI patients have been observed [5–7]. In the Traumatic Brain Injury-Practice Based Evidence study, it was determined that variations in care among TBIMS centers are due to differences in hospital characteristics, patient characteristics, and the experience of the clinicians [6–8]. Differential patient outcomes across inpatient TBIMS centers may also relate to processes of care (e.g., goal and treatment planning, selection of therapeutic interventions) [5, 6, 9]. These variations in care may result from suboptimal adoption of evidence-based best practices in routine clinical care, with a little over half of adult patients in the U.S. receiving recommended preventative, acute, and chronic disease care [10, 11]. Similar gaps in the acute management of patients at Level 1 trauma centers also exists for people following TBI [11, 12]. Although it has been shown that improved compliance with recommended care is associated with improved outcomes following TBI in the trauma setting, compliance with the limited evidence-based guidelines available in the rehabilitation milieu remains suboptimal [11, 12].

To further compound lack of a uniform approach to rehabilitation, the current literature supporting care practices in TBI rehabilitation does not allow for the identification of evidence-based care guidelines for optimal dose or intensity of therapy, the ideal timing of therapy in the recovery process, or the necessary modifications for subpopulations. This is due to variability in care practices and a lack of rigorously executed research including randomized controlled trials [6, 9, 13]. Thus, there is a critical need to resolve the gaps that exist in generating TBI clinical care guidelines. In order to address this gap, we formed a multidisciplinary group of clinicians and researchers to identify and evaluate evidence-based best practices for patients with TBI undergoing rehabilitation. The manuscript describes the process we have developed, so that it may be replicated by other groups as practitioners and researchers in the field of TBI continue to strengthen the knowledge base.

## Methods – Fig. 1

**Fig. 1:**
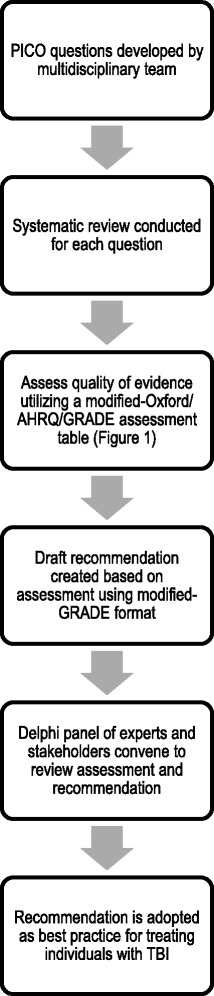
Recommendation development process PICO indicates Population, Intervention, Comparison, Outcome; AHRQ, Agency for Healthcare Research and Quality; GRADE, Grading of Recommendations, Assessment, Development and Evaluation; TBI, traumatic brain injury

### Develop PICO questions

A team of research personnel and multidisciplinary clinicians who specialize in treating patients with TBI was established including representatives from the following disciplines: physiatry, neuropsychology, physical therapy, occupational therapy, speech language pathology, and therapeutic recreation. Clinicians created a list of keywords specific to their discipline based on common clinical problems or symptoms that they treat in their day-to-day practices. For example, key words chosen by physical therapists included hemiparesis, spasticity, balance, and ataxia. Research personnel then conducted a preliminary literature search using the keywords combined with “traumatic brain injury” to identify existing literature. Based on the initial evidence collected, clinicians were then asked to form research questions in PICO format (Population, Intervention, Comparison, Outcome) related to their clinical discipline and TBI rehabilitation interests (e.g., “In patients with traumatic brain injury, does prolonged positioning such as casting versus other therapies improve spasticity?”). The goal of this exercise was to generate a list of PICO questions in order to evaluate current literature and develop evidence-based recommendations.

### Systematic review

We conducted a systematic literature review for each individual PICO question using search terms identified by the clinician in conjunction with the corresponding “brain injury” medical subject heading. PubMed, CINAHL, PSYCInfo, and Allied Health Evidence databases were utilized because they were projected to include the most content related to TBI rehabilitation research across clinical disciplines. Inclusion criteria for articles were limited to clinical trials, randomized controlled trials, comparative studies, observational studies, and case-series. Meta-analyses were also reviewed for relevant papers. Articles were included if they pertained to adults (≥18 years of age) with TBI and were published in English from January 1, 1987 – current date. Additionally, studies with a patient population of mild through severe and acute and chronic TBIs were considered. Relevant articles cited within manuscripts were also reviewed to find additional evidence to support the PICO question. Finally, articles were collated and sent to the clinician to review for applicability.

### Quality of evidence

After identifying relevant literature for PICO questions, the next step was to assess the quality of evidence for the purpose of developing clinical practice recommendations [14]. Research personnel were trained to use the Grading of Recommendations, Assessment, Development and Evaluation (GRADE) methodology to evaluate the quality of studies pertaining to each PICO question [15]. GRADE is a transparent system for rating quality of evidence of systematic reviews and journal articles and grading the strength of recommendations of guidelines [16]. However, the group found it difficult to apply GRADE methodology to evaluate the level and quality of evidence of rehabilitation data. Specifically, the team found that the TBI rehabilitation research identified through the systematic review did not meet GRADE criteria because of the variations in design of the clinical trials, variation in outcome measures, and/or heterogeneous patient populations (i.e., other acquired brain injury mixed with TBI) and care settings [13]. As a result, GRADE criteria such as inconsistency, indirectness of evidence, imprecision, effect, and dose–response relationship were difficult to assess.

We then reviewed other methodologies used to generate evidence-based recommendations and developed a modified ranking methodology. This combined the level of evidence methodology from the (1) Oxford Centre for Evidence-Based Medicine 2011 Levels of Evidence [17], (2) study population applicability from Agency for Healthcare Research and Quality Methods Guide for Effectiveness and Comparative Effectiveness reviews [18], and (3) a modified quality of evidence assessment and recommendation format from GRADE methodology shown in Tables 1, 2, and 3, respectively [15–18].A modified-GRADE methodology has been used successfully to create recommendations for other neurological disabilities and kidney disease [19, 20]. Table 3 displays the modified quality of evidence assessment criteria that we used. We found this modified approach more manageable, appropriate, and pertinent to TBI literature when compared to using only the GRADE-based methodology.

**Table 1:** Oxford Centre for Evidence-Based Medicine 2011 Levels of Evidence [17]

Level	Criteria
Level 1	Systematic review of randomized trials or n-of-1 trials
Level 2	Randomized trial or observational study with dramatic effect including crossover studies
Level 3	Non-randomized controlled cohort/follow-up study
Level 4	Case-series, case–control, or historically controlled studies
Level 5	Mechanism-based reasoning

**Table 2:** Assessment of Study Applicability based on Agency for Healthcare Research and Quality Methods

Level III	Sample is representative of the entire traumatic brain injury (TBI) population or the results are applicable to the entire TBI population
Level II	Sample is representative of a relevant subgroup of the target TBI population (i.e., patients <1 year post-injury, patients <65 years of age, etc.)
Level I	Sample is only representative of a narrow subgroup of the target TBI population and not well generalizable to other subgroups

**Table 3:** Quality Assessment based on Grading of Recommendations, Assessment, Development and Evaluation (GRADE) – Modified

Good (low risk of bias)	These studies have the least bias and results are considered valid. A study that adheres mostly to the commonly held concepts of high quality including the following: a formal randomized controlled study; clear description of the population, setting, interventions, and comparison groups; appropriate measurement of outcomes; appropriate statistical and analytic methods and reporting; no reporting errors; low dropout rate; and clear reporting of dropouts.
Fair	These studies are susceptible to some bias, but it is not sufficient to invalidate the results. They do not meet all the criteria required for a rating of good quality because they have some deficiencies, but no flaw is likely to cause major bias. The study may be missing information, making it difficult to assess limitations and potential problems.
Poor (high risk of bias)	These studies have significant flaws that imply biases of various types that may invalidate the results. They have serious errors in design, analysis, or reporting; large amounts of missing information; or discrepancies in reporting.

The multidisciplinary team assessed the quality of evidence for one PICO question at a time. The team members individually reviewed the articles and completed the quality of evidence assessment table. Afterwards, the clinician who developed the PICO question led the discussion, and as a group, level of evidence, applicability, and quality of evidence were discussed and scored for each article. Additionally, footnotes were produced to indicate the rationale for how the quality of the articles was assessed (e.g., potential confounding treatment effects or risk of bias that lowered quality of evidence).

### Delphi panel and making a recommendation

IRB approval was obtained to recruit Delphi panel members consisting of clinical professionals who treat patients with TBI and stakeholders (i.e., patients, caregivers) with a waiver of informed consent. Panel members were recruited through TBI professional organizational membership rosters for each disciplinary field via email invitation. Additionally, TBI survivors and their family members who participate in our local TBI advisory council were recruited to gauge the relevance of the research for real-world application.

## Results

### Quality of evidence

Based on the results of the quality of evidence assessment table, a draft recommendation was created using GRADE recommendation formatting (e.g., weak/conditional in favor of prolonged positioning to improve spasticity in patients with TBI undergoing rehabilitation). Table 4 shows the GRADE grid for the strength of the recommendation. The draft recommendation was then posed to the Delphi panel to achieve consensus.

**Table 4:** Grading of Recommendations, Assessment, Development and Evaluation (GRADE) grid for the strength of the recommendation

GRADE strength	Strong	Weak/conditional	Exception	Weak/conditional	Strong
Assessors’ view of the balance of desirable and undesirable consequences of the intervention	Desirable consequences clearly outweigh undesirable consequences	Desirable consequences probably outweigh undesirable consequences		Undesirable consequences probably outweigh desirable consequences	Undesirable consequences clearly outweigh desirable consequences
Recommendation	We recommend to “do something”	We suggest/conditionally recommend to “do something”		We suggest/conditionally recommend to “not do something”	We recommend to “not do something”

### Delphi panel and making a recommendation

**Fig. 2:**
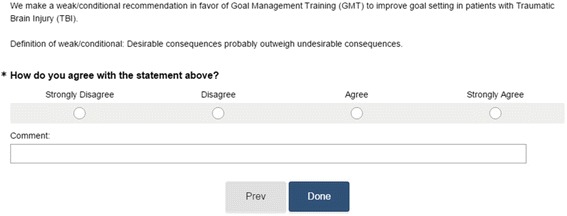
Example of Delphi panel Likert survey

The quality of evidence assessment table and corresponding articles were shared with Delphi panel members for review. However, footnotes and additional qualifying information by our group of clinicians was excluded to avoid biasing Delphi panel members. A conference call was then held to discuss the impetus of the study, history regarding the TBIMS, background information regarding the quality of evidence classification schemes employed in this study, and the process of draft recommendation development. Panel members were then directed to a HIPAA compliant web-based survey tool where they rated on a 4-point Likert scale the degree to which they agree with the draft recommendation (Figure 2). If 70% of the panelists agreed (to accept or reject the draft recommendation), then participation was complete, and the recommendation was finalized and disseminated via email back to the panel with manuscript publication to follow. Results and feedback were collated and sent to panel members via email. If 70% of panelists did not agree, panel members were asked to review the results and feedback of the first vote, and re-rate their agreement or disagreement with the draft recommendation based on the new information. Following the second round of rating (if necessary), participation was complete. If a 70% agreement was achieved following the second round of ratings, then the recommendation was finalized disseminated. If agreement was not reached, this finding was still reported. The implication of an inability to reach agreement was that the quality of evidence for that particular clinical (PICO) question is inconclusive, which suggests that further research is required.

## Discussion

Developing evidence-based clinical practice guidelines is critical to improving quality of care and rehabilitation outcomes for TBI survivors. The process described in this paper provides a comprehensive approach to addressing the issue. However, there were several constraints that were noted throughout the process that warrant discussion as a means of refining the process. First, several attempts were made trying to utilize GRADE methodology in its original form for each PICO question before the quality of evidence review process was reorganized. After reorganization, all PICO questions that were reviewed under GRADE methodology were reevaluated using the modified ranking methodology. In addition, due to a lack of research articles for many of the original PICO questions created, several meetings were dedicated to generating new PICO questions. Furthermore, it was difficult for clinical staff to fit literature reviews and meetings times related to the recommendation process into their clinical load. Likewise, the time and process to recruit Delphi panel members was also challenging given personal and professional demands on time.

While GRADE is deemed the gold standard for evaluating evidence and generating recommendations, it was not applicable for TBI rehabilitation research due to the lack of published TBI rehabilitation articles that had used robust methodologies (e.g., randomized controlled trials) within each discipline of rehabilitation [13]. In addition, outcomes in rehabilitation were not as concrete as the mortality and morbidity outcomes in medical literature, with GRADE appearing to be more relevant to medical and pharma-related outcomes. Despite the initial challenges of using GRADE methodology, the modified GRADE-Oxford did account for the unique design, outcome, and injury-related factors inherent to TBI rehabilitation research, thereby enabling this group to develop clinically relevant recommendations. By combining classification schemes into a unified quality evaluation tool, we were able to create a standardized process allowing for the aggregate review of heterogeneous studies (e.g., combination of randomized controlled trials and observational studies) with small patient populations and variations in outcome measures.

## Conclusion

There is a need for more evidence-based practice guidelines to improve quality of rehabilitation care for TBI survivors. Recommendations were created across physiatry; neuropsychology; and speech, physical, occupational, and recreational therapy disciplines using the process described above. In creating the process, it became evident that there is a need for higher-quality research for individuals with TBI in the rehabilitation setting. Additionally, it is important to develop and standardize utilization of common data elements and outcome measures for research studies to enable researchers to more adequately compare different therapies and identify best practices. Finally, it is valuable and necessary to include the input of TBI survivors and family members when selecting the best therapies to treat TBI sequelae and help survivors achieve their goals [13].

## Abbreviations

AHRQ: Agency for Healthcare Research and Quality
GRADE: Grading of Recommendations, Assessment, Development, and Evaluation
HIPAA: Health Insurance Portability and Accountability Act
IRB: Institutional Review Board
PICO: Population, Intervention, Control, Outcomes
TBI: traumatic brain injury
TBIMS: Traumatic Brain Injury Model System
